# Supplementary material for: Identification and Characterization of a New Type III Polyketide Synthase from a Marine Yeast, Naganishia uzbekistanensis
Source: Mar Drugs. 2020 Dec 11;18(12):637. doi: 10.3390/md18120637 (PMC7763939; doi:10.3390/md18120637)
Supplement: Supplementary file 1 [file marinedrugs-18-00637-s001.pdf]

Supplementary Materials

Identification and Characterization of a New Type III Polyketide Synthase from a Marine Yeast, *Naganishia uzbekistanensis*

Laure Martinelli [1,†,‡], Vanessa Redou [1,‡], Bastien Cochereau [1], Ludovic Delage [2], Nolwenn Hymery [1], Elisabeth Poirier [1], Christophe Le Meur [1], Gaetan Le Foch [1], Lionel Cladiere [2], Mohamed Mehiri [3], Nathalie Demont-Caulet [4] and Laurence Meslet-Cladiere [1,*]

[1] Laboratoire Universitaire de Biodiversité et Ecologie Microbienne, University Brest, F-29280 Plouzané, France; laure.martinelli@univ-st-etienne.fr (L.M.); vanessa.redou@gmail.com (V.R.); bastien.cochereau@etu.univ-nantes.fr (B.C.); nolwenn.hymery@univ-brest.fr (N.H.); Elisabeth.Poirier@univ-brest.fr (E.P.); christophe.lemeur@univ-brest.fr (C.L.M.); Gaetan.lefloch@univ-brest.fr (G.L.F.)

[2] Integrative Biology of Marine Models (LBI2M), Station Biologique de Roscoff (SBR),CNRS, UMR8227, Sorbonne Université, 29680 Roscoff, France; delage@sb-roscoff.fr (L.D.); cladiere@sb-roscoff.fr (L.C.)

[3] Marine Natural Products Team, CNRS, UMR 7272, Institut de Chimie de Nice, Université Côte d'Azur, 06108, Nice, France; mohamed.mehiri@unice.fr

[4] UMR ECOSYS, INRAE, INRAE, University of Paris, AgroParisTech, Université Paris-Saclay, 78026, Versailles, France; nathalie.demont-caulet@inra.fr

* Correspondence: Laurence.meslet@univ-brest.fr; Tel.: +33-2-90-91-51-10

† Present address: Laboratoire BVpam—FRE, Université de Lyon, UJM-Saint-Etienne, CNRS, 3727, Saint-Étienne, France.

‡ These authors contributed equally to this work.

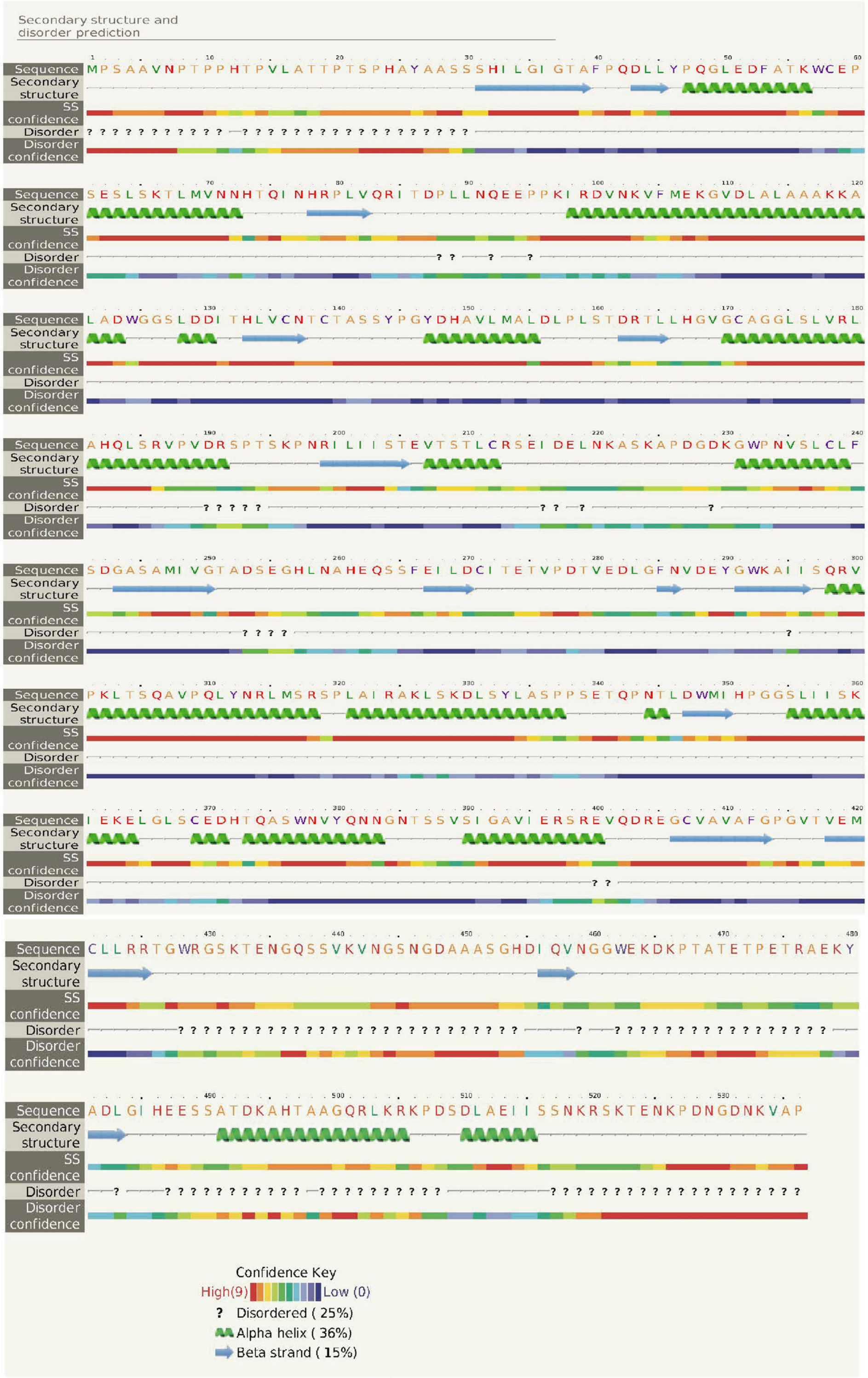

Figure S1. Secondary structure of PKSIII Mo29 and disorder prediction by Phyre2 [48].

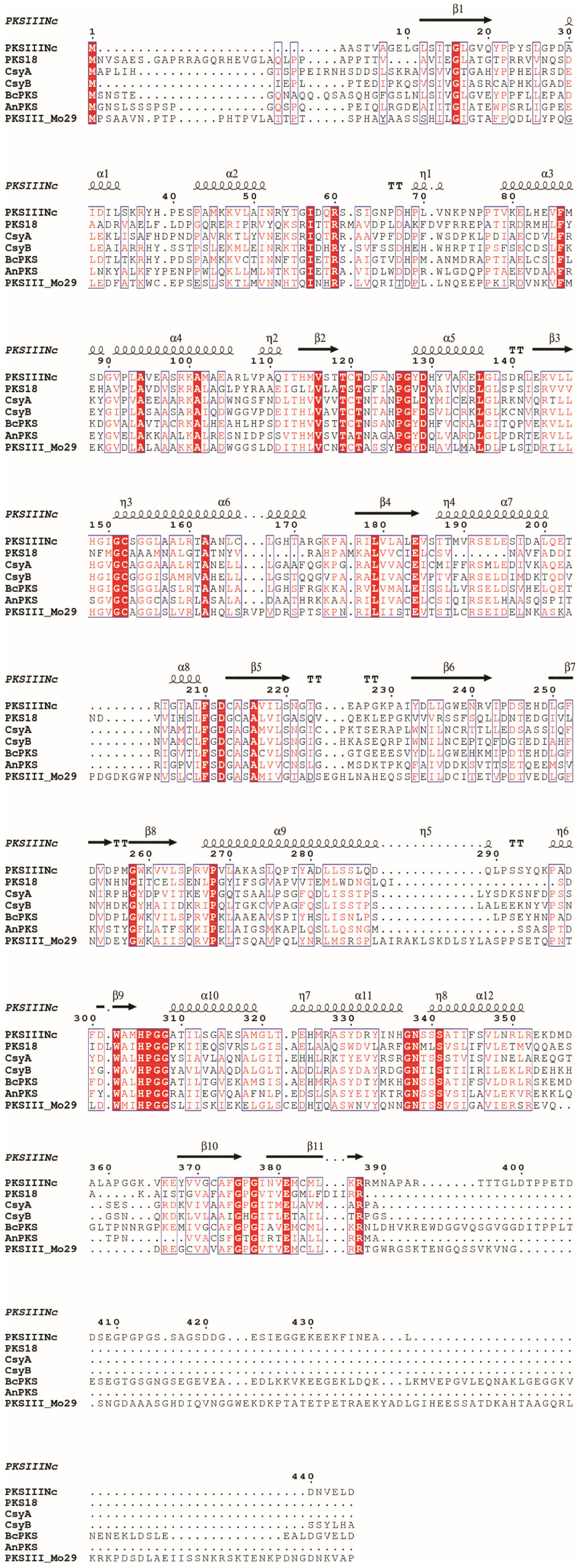

Figure S2. Amino acids sequence alignment of PKSIII Mo29 with PKSIII from *Neurospora crassa* (PKSIIINc), from *Mycobacterium tuberculosis* (PKS18), from *Aspergillus oryzae* (AoCsyA and AoCsyB), from *Botrytis cinerea* (BcPKS) and *Aspergillus niger* (AnPKSIII) [37–39,41,51,53].
